# Different clinical presentation and management of temporal bone fibrous dysplasia in children

**DOI:** 10.1186/s12957-017-1302-5

**Published:** 2018-01-15

**Authors:** Józef Mierzwiński, Justyna Kosowska, Justyna Tyra, Karolina Haber, Maria Drela, Dariusz Paczkowski, Paweł Burduk

**Affiliations:** 1 Department of Otolaryngology, Audiology and Phoniatrics, Children’s Hospital of Bydgoszcz, Chodkiewicza 44, 85-667 Bydgoszcz, Poland; 2 Department of Pediatric Neurosurgery, Children’s Hospital of Bydgoszcz, Chodkiewicza 44, 85-667 Bydgoszcz, Poland; 3 Department of Otolaryngology and Laryngological Oncology Faculty of Medicine, Collegium Medicum Nicolaus Copernicus University, Bydgoszcz, Poland

**Keywords:** Fibrous dysplasia, Temporal bone, Child

## Abstract

**Background:**

Fibrous dysplasia is a slowly progressive benign fibro-osseous disorder that involves one or multiple bones with a unilateral distribution in most cases. It is a lesion of unknown etiology, uncertain pathogenesis, and diverse histopathology. Temporal bone involvement is the least frequently reported type, especially in children.

We reviewed available articles regarding fibrous dysplasia with temporal bone involvement in children and added four patients aged 7 to 17 years who were diagnosed and treated in our institution from 2006 to 2017. The patients’ clinical picture comprised head deformity, external canal stenosis, headache, progressive conductive and/or sensorineural hearing loss, tinnitus, and sudden deafness. Two patients had experienced severe episodic vertigo with nausea and vomiting. Two were referred to us with external canal obstruction and secondary cholesteatoma formation with broad middle ear destruction. One was diagnosed with acute mastoiditis and intracranial complications. Optimal management of fibrous dysplasia is unclear and can be challenging, especially in children. In our two patients with disease expansion and involvement of important structures, surgical treatment was abandoned and a “wait-and-scan” policy was applied. The other two were qualified for surgical treatment. One patient underwent two surgeries: modified lateral petrosectomy (canal left open) with pathological tissue removal, cavity obliteration, and subsequent tympanoplasty. Another patient with extensive destruction of the left temporal bone underwent canal wall down mastoidectomy with perisinus abscess drainage and revision 12 months later. Tympanoplasty was unsatisfactory in both patients because of slow progression of the middle ear pathology. None of our patients underwent pharmacological treatment.

**Conclusions:**

In younger patients, observation and a “wait-and-scan” protocol is relevant until significant function, or cosmetic deficits are obvious. Surgery is not preferred and should be delayed until puberty because fibrous dysplasia has a tendency to stabilize after adolescence. In patients with severe symptoms medical treatment can be implemented, but safety of this treatment in children remain controversial.

## Background

Fibrous dysplasia (FD) is a rare benign but progressive bone disorder. The main pathological change in FD is slow replacement of the normal bone structure with fibrous tissue. Formation of pathological tissue can lead to deformities, pathological fractures, and symptoms related to the anatomic location of the pathology such as cranial nerve deficits and hearing and vision loss [1–4]. FD may occur at any time and sometimes develops in older people; however, it typically occurs before the age of 30 years [5]. FD accounts for 2.5% of all osseous neoplasms and 7.0% of all benign bone tumors [6, 7].

FD was first described in 1938 by Lichtenstein [8]. The author suggested that FD is caused by a defect in the differentiation of the mesenchymal bone precursor. In 1957, Changus [9] stated that fibroblast hyperplasia plays a crucial role in the pathogenesis of FD. Actual data based on genetic and molecular analyses suggest that pathological regulation of intercellular cAMP or protein kinase dysfunction contributes to FD. Other studies revealed a somatic activating mutation within the *GNAS1* gene located on chromosome 20 [6, 10]. In 2015, Pardo-Maza et al. [11] described a case of FD in which the pathologic process was suggested to be a late complication of temporal bone cholesteatoma surgery.

FD can involve any bone in the body and is classified according to the localization of the pathological changes. The most common type is the monostotic form, which accounts for about 70% of all cases of FD and is characterized by the involvement of only one bone. The next most common type is the polyostotic form, which accounts for about 30% of cases and involves more than one bone [1, 12, 13]. The most severe type of FD is characterized by whole-body involvement; this type of FD is called McCune–Albright syndrome and affects 3% of patients. It involves both the bones and endocrine system and is associated with café-au-lait spots and various endocrine disorders including premature puberty, hyperthyroidism, acromegaly, Cushing’s syndrome, and hyperparathyroidism. Cardiovascular and digestive system abnormalities may also develop [6, 12, 14].

FD shows a racial predilection in that Caucasians account for > 80% of affected patients while Asians account for only 1% [12]. FD involving only the temporal bone was described for the first time in 1946 by Schlumberger [15]. Since then, more than 100 cases of FD of the temporal bone have been described, including 66 cases in children [12, 13].

FD is a progressive, slowly developing condition with tendency to regress after puberty, and the optimal treatment thus remains unclear in many cases. A recent report recommended surgical treatment after puberty [7].

## Materials and methods

We analyzed the number of children diagnosed with temporal bone FD treated or observed from 2006 to 2017 in our department. Four patients with isolated FD of the temporal bone were treated in our institution.

A systematic review of all articles published prior to December 2017 was performed through a search of the PubMed electronic database. The investigation strategy was based on an advanced search with the following additional filters: English language, age of birth to 18 years, and inclusion of the text words (in all fields) temporal bone and temporal area combined with the operator AND and the text word fibrous dysplasia (in all fields).

We reviewed publications describing children with FD involving the temporal bone and excluded articles describing patients aged > 18 years and those in which the age group was not identified. Some reports that were missed using this strategy were identified from the reference lists of the analyzed papers. Moreover, in 1995, Megerian et al. [12] reviewed the English-language literature to identify cases of FD of the temporal bone. Children from reports not listed in PubMed but reviewed by Megerian et al. [12] were additionally included.

The abovementioned criteria revealed 147 English-language studies; the additional age filter for children provided 58 search results. The full text of all articles was assessed. Thirty reports of 49 children with FD involving the temporal bone were found. Three articles involving seven children were added after screening the references. Ten children from 10 articles in the review by Megerian et al. were also added.

Finally, we identified 66 children with FD involving the temporal bone reported since 1950 in the English language literature.

## Results

### Literature review results are presented in the Table 1

Review included 66 children with FD involving the temporal bone reported in the 43 English language studies. The mean age of presentation was 12 years (SD. 4.4), ranging from 9 months to 17 years. There were 32 boys (48.5%), 26 girls (39.4%); sex was not reported in 8 cases (12.1%). The clinical pattern of FD was monostotic form in 43 cases (65%), polyostotic form in 19 cases (29%), the most severe type—McCune–Albright syndrome was diagnosed in 4 patients and accounted for 6%.

**Table 1:** Previously published reports of temporal bone FD in children

Author	No.	Age	Sex	Symptoms	Form	Treatment
Present study	1	7	M	Vertigo, nausea, vomiting, tinittus, headache, local swelling	Monostotic	Nonsurgical
	2	16	F	Headaches, skull asymmetry, HL, mass, EAC stenosis	Monostotic	Modified lateral petrosectomy with complete removal of the lesion
	3	13	F	HL, vertigo, tinittus, vomiting, visual disorder	Polyostotic	Nonsurgical
	4	16	M	Mastoiditis, HL, EAC stenosis, otalgia, otorrhea, mass, left ear cholesteatoma	Monostotic	Mastoidectomy, perisinus abscess drainage, revision
Previously published reports of temporal bone FD in children						
Articles added after screening the references						
Celenk et al. [34]	1	17	F	Painless, unilateral mandibular swelling	Polyostotic	Nonsurgical-periodic follow up
Fattah et al. [7]	2	6	–	Facial asymmetry, malocclusion	Monostotic	No data
	3	6	–	Mass	Monostotic	No data
	4	17	–	Mass, EAC stenosis	Monostotic	No data
	5	14	–	Mass	Polyostotic	No data
	6	8	–	Snoring, epistaxis, deteriorating acuity;	Polyostotic	No data
Couturier et al. [6]	7	10	M	EAC stenosis (normal hearing)	Monostotic	Canaloplasty, osseointegrated implant
Reports not listed in PubMed reviewed by Megerian						
Megerian et al. [12]	8	5	F	Vertigo, SNHL, FN paralysis	Monostotic	Nonsurgical
	9	7	F	HL, EAC stenosis	Monostotic	Nonsurgical
	10	16	M	Mass	Monostotic	Mastoid surgery
	11	3	M	Otitis, EAC stenosis, SNHL, EAC cholesteatoma	Polyostotic	Mastoidectomy, canaloplasty
	12	17	F	HL, EAC stenosis	Monostotic	Canaloplasty, mastoidectomy
	13	11	M	HL	Monostotic	Canaloplasty, mastoidectomy
	14	11	M	HL, mass, EAC stenosis, EAC cholesteatoma, FN palsy	Monostotic	Mastoid surgery
	15	9	M	HL, EAC stenosis	Polyostotic	Canaloplasty
	16	16	F	HL, otalgia, EAC stenosis	Monostotic	Nonsurgical
	17	14	M	HL, EAC stenosis	Monostotic	Mastoid surgery
Articles listed in PubMed						
Pardo-Maza et al. [11]	18	16	F	Recurrent suppurative otitis media, HL	Monostotic	Mastoidectomy, A surgical revision of the ear cavity, resecting the lesion and regularizing the cavity
Du et al. [35]	19	16	M	Painless vision loss in both eyes	Polyostotic	Nonsurgical
Shakeel et al. [36]	20	16	M	Painless swelling—temporal area, no other symptoms	Polyostotic	Affected temporal bone was resected, and gross total removal was achieved
Yang et al. [37]	21	15	F	HL, EAC stenosis, EAC cholesteatoma	Monostotic	Canaloplasty
Cai et al. [38]	22	15	M	Local lump, facial malformation	Monostotic	Surgical resection
	23	6	M	Atypical headaches	Polyostotic	Surgical resection
	24	15	F	Atypical headaches, proptosis	Polyostotic	Complete removal and reconstruction with a titanium allegation implant
	25	11	M	Local lump	Monostotic	Complete removal and reconstruction with a titanium implant
	26	14	F	Local lump, HL	Polyostotic	Complete removal and reconstruction with a titanium implant
Jethanamest et al. [39]	27	17	M	HL, EAC atresia, cholesteatoma	Monostotic	Mastoidectomy, canaloplasty
Kim et al. [13]	28	17	F	HL, EAC stenosis	Monostotic	Mastoidectomy, canaloplasty
	29	13	M	HL, EAC stenosis, cholesteatoma	Monostotic	Mastoidectomy, canaloplasty
	30	16	M	HL, EAC stenosis	Monostotic	Mastoidectomy, canaloplasty
	31	14	M	EAC stenosis, ear fullness	Monostotic	Canaloplasty
	32	14	M	Sudden HL-fluctuating SNHL, vertigo	Monostotic	Transmastoid labyrinthectomy
Keskin et al. [40]	33	16	F	Painful facial swelling-mandible	Polyostotic	Segmental mandibulectomy, mass resection
Martinez et al. [20]	34	7	M	HL, EAC stenosis, mass, proptosis, trismus, bleeding, head pain	Polyostotic	Nonsurgical-medical therapy, EAC debris removed
Sreetharan et al. [41]	35	16	M	Postauricular swelling, EAC stenosis	Monostotic	Nonsurgical-managed conservatively
Tweddle et al. [42]	36	16	M	Recurrent otitis externa, tinnitus, EAC stenosis, normal hearing	Monostotic	Mastoidectomy, EAC reconstruction
Ozbek et al. [43]	37	10	F	HL, postauricular swelling, recurrent otitis externa, EAC stenosis	Monostotic	Nonsurgical-periodic CT scanning
Magu et al. [44]	38	15	F	Headache, facial asymmetry–FN palsy	Polyostotic	No data
Lustig et al. [45]	39	15	–	HL, EAC stenosis	McCune–Albright syndrome	Canalplasty
	40	11	–	Proptosis	Polyostotic	Surgical drainage, cystoethmoidistomy, pericranial closure
	41	17	–	Headache	Monostotic	Middle fossa mass excision
Chinski et al. [25]	42	16	M	HL, EAC stenosis	Monostotic	Nonsurgical-periodically examined
Ohta et al. [46]	43	16	M	Tumor	Monostotic	Surgical
Megerian et al. [12]	44	4	M	Tumor, HL, otalgia, otorrhea, EAC stenosis, recurrent otitis externa	Monostotic	Nonsurgical
	45	9	F	HL, EAC stenosis, otalgia, otitis externa	Monostotic	Surgical, infratemporal approach
	46	12	F	HL, EAC stenosis, canal cholesteatoma, aural pressure, facial asymmetry	Polyostotic	Mastoidectomy, canaloplasty
	47	12	F	HL, EAC stenosis, trismus, otalgia, otorrhea	Monostotic	Canaloplasty TMJ arthroplasty
	48	5	F	HL, EAC stenosis, mass	Polyostotic	Conservative
	49	10	F	HL, EAC stenosis, frontal region protrusion, aural fullness, facial asymmetry, otitis externa	Polyostotic	Surgical-maxilla
	50	2	M	EAC stenosis, HL in audio, FN paralysis	Monostotic	Mastoidectomy, canaloplasty, tympanoplasty, FN decompression
Mizuno et al. [47]	51	16	F	Temporal pain, temporal area deformation, malocclusion	McCune–Albright syndrome	Surgical-mandible
Kessler et al. [48]	52	15	M	HL, EAC stenosis	Monostotic	Nonsurgical
Talmi et al. [49]	53	15	F	Recurrent otitis externa, HL, EAC stenosis, ear fullness	Monostotic	Surgical, canaloplasty
Younus et al. [50]	54	9/12	M	Mass, EAC stenosis	Monostotic	Mastoidectomy, canaloplasty
Smoucha et al. [51]	55	14	M	HL, EAC stenosis, mass, otitis externa	Monostotic	Mastoidectomy, canaloplasty
Sataloff et al. [52]	56	9	F	HL, EAC stenosis	Polyostotic	Mastoidectomy, canalplasty
Nishioka et al. [53]	57	11	M	Epileptic attacks, mass, EAC stenosis	Monostotic	No data
Nager et al. [54]	58	10	M	HL, EAC stenosis, EAC cholesteatoma	McCune–Albright syndrome	Canaloplasty
	59	10	M	HL, EAC stenosis	Monostotic	PE tube
Barrionuevo et al. [17]	60	11	F	Mass, otalgia	Monostotic	Nonsurgical
	61	3	F	Premature puberty	McCune–Albright syndrome	Nonsurgical
Williams et al. [55]	62	10	F	HL, EAC stenosis	Monostotic	Mastoidectomy, canalooplasty
Talbot et al. [56]	63	12	M	HL, EAC stenosis	Monostotic	Canaloplasty
Cohen et al. [57]	64	8	F	Mass, EAC stenosis, FN paralysis, SNHL	Polyostotic	Mastoid surgery
	65	9	M	Mass, EAC stenosis	Monostotic	Mastoidectomy, canaloplasty
Wong et al. [58]	66	13	F	HL, mass, EAC stenosis, EAC cholesteatoma	Monostotic	Canaloplasty

*HL* hearing loss, *SNHL* sensorineural hearing loss, *EAC* external auditory canal, *FN* facial nerve

The presenting symptoms included hearing loss (58%), tumor or deformity (38%), otitis media, externa or recurrent otitis externa (14%), facial nerve palsy (12%), otorrhea or otalgia (11%), headaches (8%), ear fullness (6%), proptosis (5%), trismus (3%), vertigo (3%), and tinnitus (2%). Other rarely reported symptoms include epistaxis, deteriorating acuity, epileptic attacks, malocclusion, premature puberty. The most frequent finding was EAC stenosis that occurred in 62% of children. Cholesteatoma of external auditory canal was diagnosed in 12% of patients. Sudden deafness was described for the first time in our study.

Sixty-eight percent of patients (45 children) underwent surgical intervention, in 21% (14 cases) nonsurgical treatment appeared to be effective. Treatment modality was impossible to establish for seven patients. In 25 (38%) patients, canaloplasty was performed. Twenty (30%) patients underwent mastoidectomy or surgical removal of the lesion involving mastoid process. Ten (15%) patients were treated with other complete surgical resections of the pathology. Four patients had surgery involving maxilla or mandible. One patient with FD in left temporal bone was fitted with left osseointegrated implant.

### Case 1—7-year-old boy with tinnitus and vertigo

#### Anamnesis

A 7-year-old boy was admitted to our hospital because of recurrent vertigo and balance disorders with nausea, vomiting, and intermittent tinnitus in the right ear. Additionally, he experienced a periodic moderate headache.

#### Physical examination

Examination revealed a painless swelling over the right mastoid region and occipital asymmetry on the right side. No nystagmus was present, and during the Romberg test the patient fell to the right side. The fistula test was negative. Videonystagmography revealed hyporeflection of the right vestibule (45%) as well as left directional preponderance (25%). An audiologic evaluation demonstrated normal hearing.

**Fig. 1:**
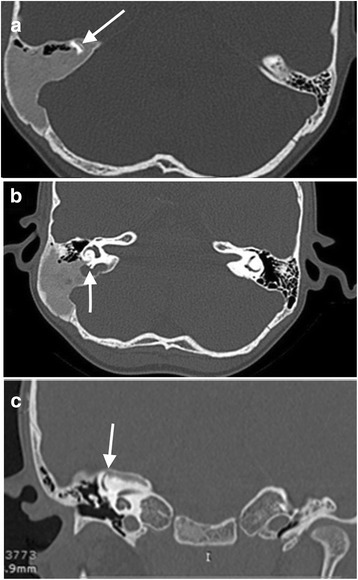
**a** Coronal CT scan shows involvement of the petrous part of the right temporal bone. The superior semicircular canal is surrounded by dysplasia. **b** Axial CT scan shows erosion of the labyrinth and involvement of the posterior semicircular canal. **c** Axial CT scan shows massive changes in the petrous part and a fistula of the superior semicircular canal

Imaging findings (Fig. 1)

- Treatment: We decided not to perform surgery because of considerable expansion of the FD in the skull base and risk of vestibular damage.
- Clinical course of the disease: The vertigo recurred throughout the 3-year follow-up period. Gradual deterioration in vestibular function was observed (35–65%). 3 years after the first hospitalization, the patient was again admitted to our hospital because of an episode of sudden deafness. His hearing gradually returned to normal after intravenous steroid treatment.

### Case 2—16-year-old girl with conductive hearing loss and skull asymmetry

#### Anamnesis

A 16-year-old girl was admitted to the neurology department because of severe headaches and skull asymmetry. The patient had begun to notice hearing loss approximately 1 year earlier. An audiogram at that time demonstrated conductive hearing loss in the right ear; the air-bone gap was 60 dB.

#### Physical examination

Clinical examination additionally revealed a large mass over the right temporal area and obstruction of the right external auditory canal.

#### Imaging findings

**Fig. 2:**
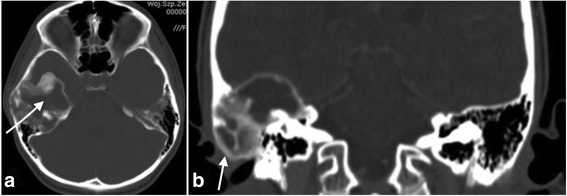
**a** Axial CT scan shows an expansive lesion of the right petrous apex. **b** Coronal CT scan demonstrates fibrous dysplasia of the right temporal bone that has led to external deformity

**Fig. 3:**
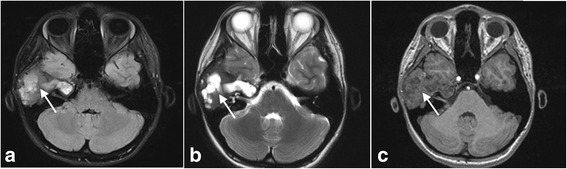
**a** Magnetic resonance imaging. T1-weighted image. **b** T2-weighted image. **c** Contrast-enhanced T1-weighted image shows a heterogeneous mass with contrast enhancement. The radiographic features of the lesion did not allow an experienced radiologist to establish the diagnosis

**Fig. 4:**
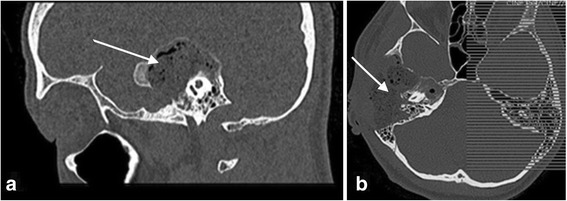
**a** Postoperative sagittal CT scan shows a large cavity after petrosectomy obliterated with the temporalis muscle. **b** Axial CT scan shows a large cavity after petrosectomy in the right temporal bone

Magnetic resonance imaging (MRI) and computed tomography (CT) were performed. Imaging studies showed a large, extensive, and heterogeneous mass over the right temporal area (Figs. 2, 3, and 4).These findings initially suggested a temporal bone tumor of unknown origin.

#### Treatment

To ensure the best outcome, and considering all of the patient’s symptoms including severe headaches, hearing loss, and cranial deformity, surgical treatment was performed by a multidisciplinary team that included a neurosurgeon and otolaryngologist. Intraoperatively, the severely bleeding mass of the temporal bone was suspected to be a tumor. The lesion involved almost the whole temporal bone and extended from the mastoid process to the petrous apex. There was no involvement of the otic capsule. Middle ear exploration revealed a completely obliterated external auditory canal and involvement of the ossicular chain with immobilization. The patient underwent modified petrosectomy with complete removal of the degenerative temporal bone lesion (Fig. 4). In spite of the broad surgery and tissue removal, we did not perform blind-sac closure of the ear in an attempt to improve and preserve the patient’s hearing. After partial obliteration of the surgical cavity with a rotated temporalis muscle flap, the external auditory canal was left open and tympanoplasty was performed with mobilization of the ossicular chain and incus interposition. Tissue specimens taken during surgery were sent out for histopathological examination which revealed FD.

#### Clinical course of the disease

Postoperative CT and MRI were performed with audiometric testing. Some improvement in hearing was observed initially, but with the slow progression of the disease, the patient’s hearing deteriorated and she underwent a second surgery with tympanoplasty. After an initial improvement, her hearing gradually deteriorated to 45 dB (the air-bone gap at 0.5–4.0 kHz) again. The patient was observed for 5 years, and at the time of this writing, MRI demonstrated slow disease progression with a tendency toward stabilization. She complains of periodic moderate headaches.

### Case 3—13-year-old girl with sensorineural hearing loss, tinnitus, and vertigo

#### Anamnesis

A 13-year-old girl was admitted to our department with left ear hearing impairment, paroxysmal vertigo, tinnitus, vomiting, and episodes of visual disorders. Her medical history included numerous previous hospitalizations due to these symptoms.

#### Clinical examination

On clinical examination, the Romberg test was positive; the patient fell toward the left. No spontaneous nystagmus was observed. The patient reported improvement after pharmacological treatment. Videonystagmography after caloric stimulation showed hyporeflection of the left vestibule. An audiogram revealed normal hearing in the right ear and sensorineural hearing loss in the left ear with 30 dB loss (0.5–4.0 kHz bone conduction).

**Fig. 5:**
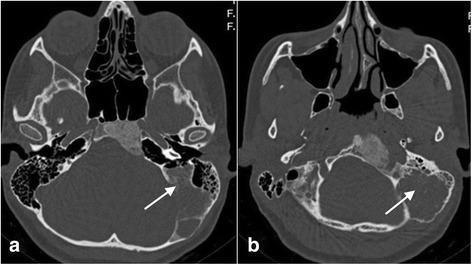
**a** Axial CT scan at the level of the cochlea demonstrates extensive involvement of the left temporal bone by fibrous dysplasia. **b** Axial CT scan at the level of the stylomastoid foramen demonstrates extensive involvement of the left temporal bone by fibrous dysplasia

**Fig. 6:**
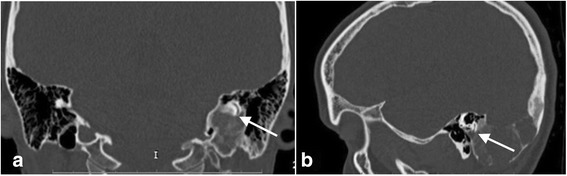
**a** Coronal CT scan shows a fistula of the posterior semicircular canal. **b** Sagittal CT scan shows extensive involvement of the petrous apex of the temporal bone by fibrous dysplasia and involvement of the labyrinth

Imaging findings (Figs. 5 and 6)

- Treatment: Considering the extent and localization of the lesion, the decision was made not to operate on the patient.
- Clinical course of the disease: Long-term follow-up of the patient showed no evidence of lesion expansion but periodic worsening of her vertigo. At the time of this writing, the patient’s hearing was stable.

### Case 4—16-year-old boy with acute mastoiditis, a left ear cholesteatoma, external auditory canal stenosis, and hearing loss

#### Anamnesis

A 16-year-old boy presented with clinical symptoms of acute mastoiditis, hearing loss in the right ear, severe otalgia, and otorrhea of 1-month duration from the affected ear.

#### Clinical examination

Physical examination revealed an inflammatory swelling of the left preauricular and postauricular areas, external auditory canal, and mastoid process. Otoscopic examination revealed marked stenosis of the external auditory canal, purulent otorrhea in large quantities, and debris in the involved ear. An audiogram demonstrated conductive hearing loss of 60 dB (the air-bone gap at 0.5–4 KHz) on the left.

**Fig. 7:**
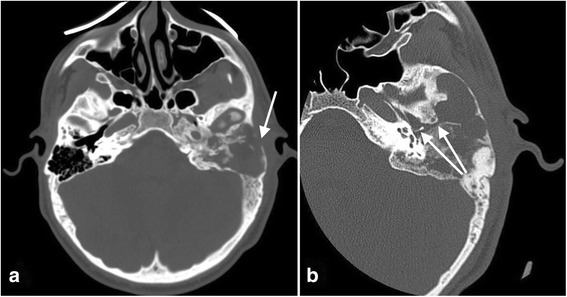
**a** Axial CT scan of the temporal bone shows massive destruction of the mastoid area with external osteolysis and a fistula in the temporal area. **b** Axial CT scan at the level of the oval window shows massive fibrous dysplasia involving the temporal bone as well as opacification of the mastoid and middle ear cavity with ossicles destruction and stenosis of the external auditory canal

**Fig. 8:**
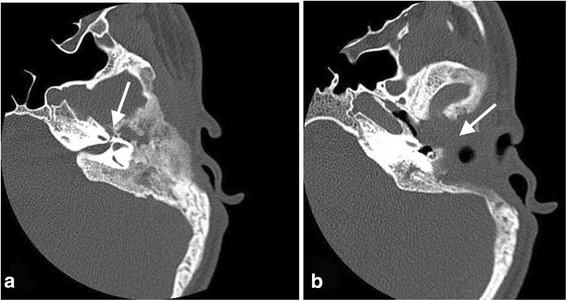
**a** Axial scan at the level of the labyrinth shows typical fibrous dysplasia-related ground-glass changes around the geniculate ganglion. **b** Axial CT scan at the level of the cochlea 1 year after surgery shows obliteration of the postoperative cavity

**Fig. 9:**
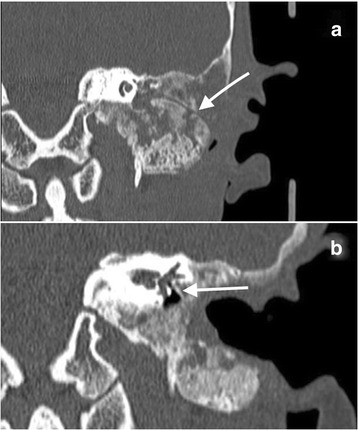
**a** Coronal scan shows obliteration of the external auditory canal with broad changes and osteolysis of the temporal bone. **b** Coronal scan post-op. shows involvement of the middle ear and stapes

Imaging findings (Figs. 7, 8, and 9)

- Treatment: The patient was immediately qualified for surgical treatment. During the surgery, we observed secondary complete occlusion of the external auditory canal and a large cholesteatoma involving the auditory canal and middle ear. Extensive destruction of the left temporal bone was noted. The patient underwent canal wall down mastoidectomy. The walls of the sigmoid sinus were thickened due to an inflammatory process and perisinus abscess. We performed surgical drainage and ossicular reconstruction (incus interposition). The diagnosis of FD initially based on the imaging findings was confirmed pathologically. The initial surgical outcome and hearing improvement were good, but within a few months, severe conductive hearing loss recurred. Repeat imaging studies demonstrated disease progression with involvement of the tympanic cavity and destruction of the sound-transferring system. Postoperative MRI showed the possibility of residual cholesteatoma. The patient underwent revision surgery 12 months after the first surgery, and the tissue that had been observed by MRI appeared to be scar tissue and silastic sheeting.
- Clinical course of the disease: Over the next 4 years of follow-up, neither recurrent cholesteatoma nor FD progression was encountered. The patient was qualified for and successfully implanted with a transcutaneous bone conduction hearing aid and was under the care of an audiologist at the time of this writing.

## Discussion

Some authors have suggested that FD is twice as common in women as in men [16, 17]. However, FD occurs with an equal sex distribution among the patients treated in our institution; this is in accordance with most reports in the world literature [6]. Review revealed a male predominance—we found 48.5% of boys and 39.4% of girls.

FD involving the temporal bone, especially in children, is very rare. In 2013, Fattah et al. [7] reported that temporal bone involvement occurred in only 3 of 37 pediatric patients with craniofacial FD. The disease is usually a slow but relentless process that may lead to serious otological manifestations. In 1980, Barrionuevo et al. [17] proposed three clinical stages of FD: stage 1, latent; stage 2, symptomatic disease; and stage 3, complication phase [17]. Although FD is a benign condition, it can be locally destructive and expansive. Moreover, the incidence of spontaneous malignant transformation reportedly ranges from 0.5 to 1.0%, and malignancies include osteosarcoma (the most common), fibrosarcoma, chondrosarcoma, and giant cell tumor [1, 13, 18]. In one study, a higher frequency of malignant transformation (4%) was observed in patients with McCune–Albright syndrome [19]. Pain, rapid lesion growth, and a dramatic elevation of alkaline phosphatase may herald malignant transformation [4, 9]. Notably, however, carcinogenesis can be induced by radiation at a reported incidence of 44% [4].

### Symptoms

The most common symptoms of pediatric temporal bone FD in descending order of frequency are stenosis of the external auditory canal (62%), hearing loss (58%), and external deformity (38%). Stenosis of the external auditory canal may result in formation of an acquired cholesteatoma and further complications, including those affecting the intracranial and intratemporal regions.

Cholesteatoma secondary to external canal narrowing occurs in 40% of patients with FD [12, 18, 20, 21]. According to presented review cholesteatoma of external auditory canal was diagnosed in 12% of children. Longstanding disease affecting the temporal bone and external auditory canal may lead to involvement of the middle and inner ear. The most frequent complications of this process are sensorineural hearing loss (14–17%) and facial nerve palsy (approximately 10%, in children with FD involving temporal bone − 12%) [4, 22]. Zaytoun et al. [22] described recurrent attacks of facial nerve paralysis that completely recovered without any sequelae. Other possible complications are otic capsule destruction and vestibular fistulization causing severe vertigo [6, 12]. The most severe and life-threatening complications involve meningitis, epidural abscesses, multiple cranial neuropathies, and massive changes involving the middle and posterior cranial fossa [4, 12, 22]. Although FD is a benign disease, it can be lethal [20].

We also observed an episode of sudden deafness in one patient; we did not find this symptom of FD in our search of the literature. Facial canal involvement was present on CT in all of our patients with FD, but facial weakness was not found.

Progressive conductive hearing loss was the most common symptom in the present study, which is comparable with the findings of other previously published studies.

Otic capsule involvement is generally rare in patients with FD, but it was present in two of four of our patients who experienced severe vertigo with nausea and vomiting due to fistulization of the labyrinth capsule [17]. One of these patients developed an episode of sudden deafness with quick recovery after steroid treatment. External canal stenosis with an acquired secondary cholesteatoma was observed in two patients and led to a serious complication (acute mastoiditis with sigmoid sinus thrombosis) in one of them. Both of these patients required prompt surgical intervention.

Headache is also a commonly described symptom in patients with craniofacial FD and occurred in three of four of the patients in our institution.

### Diagnosis

The clinical, radiologic, and histopathologic features of FD are well documented in the literature, and diagnosis has therefore become relatively easier. However, as in our second case, the imaging findings are not always typical and may be initially misdiagnosed by radiologists.

Radiographic findings of FD include thinning of cortical bone, radiolucent fibrous elements, and dark areas of sclerosis. In the literature, these imaging features are called a ground-glass appearance [23]. These characteristic radiographic features of FD were described for the first time by Fries [24] in 1957.

Three characteristic radiologic patterns of FD have been described in the literature:

- Pagetoid: This is the most common type, affecting about 56% of patients. It is characterized by areas of intermixed tissues of different density, with dense areas of sclerosis and radiolucent areas of fibrosis, resulting in a ground-glass appearance.
- Sclerotic: This is the second most common form, affecting about 23% of patients. It is characterized by homogeneous dense areas.
- Cystic: This type is rare, affecting 21% of patients. The name refers to oval or round radiolucent rings surrounded by dense bone [13, 21, 25].

CT is the most sensitive and accurate imaging modality for FD. It is the best technique with which to display bony changes and allows adequate assessment of the temporal bone. CT is useful in demonstrating all complications caused by expansile growth of FD, and special attention should be given to the inner ear structures (semicircular canals, facial nerve, and auditory ossicles). MRI might be useful in cases of cystic FD and in assessing the involvement of soft tissues [21, 25].

FD shows low signal intensity on T1-weighted MRI and varying signal intensity on T2-weighted MRI. This has been explained by the various amounts of collagen fibers inside the cells and various formation of bony trabeculae [10].

The clinical diagnosis of temporal bone FD can also be confirmed with bone scintigraphy. Bone scanning with technetium-99m is particularly useful in patients with the polyostotic form of FD. After one imaging study, it is possible to identify the extent of bone involvement in the whole body. However, we have not found it necessary to perform this type of study in our patients.

The differential diagnoses of FD include ossifying and nonossifying fibroma, osteochondroma, exostosis, osteoma, histiocytosis, Paget’s disease, aneurysmal bone cyst, unicameral bone cyst, giant cell tumor, central giant cell granuloma, sarcoma, and Brown tumor in patients with hyperparathyroidism. FD can also mimic meningioma [12, 16]. Particular difficulty may arise in differentiating ossifying fibroma from FD. Ossifying fibroma is a well-defined, focal expansile lesion with smooth margins and a tendency for aggressive growth, particularly when the lesion involves the nasal cavity, paranasal sinuses, or orbit. The diagnosis is based on radiological imaging and histopathologic examination with a characteristic clinical picture. Basically, the presence of bony lamellae and fibrous matrix confirms the diagnosis [21].

### Treatment

According to the literature, two treatment methods have traditionally been proposed. In younger patients, when the disease is limited and progression is slow, observation and a “wait-and-scan” protocol is the most commonly chosen option and is relevant until significant function, or cosmetic deficits are obvious. Surgery is not preferred and should be postponed because FD has a tendency to become inactive after puberty [13].

When the age at surgery is considered, total resection and reconstruction or debulking surgery after skeletal maturity has a lower recurrence rate (1/7 cases) than earlier surgery (8/16 cases) [7]. Complete resection at any age and debulking surgery once skeletal maturity has been reached may be associated with lower recurrence rates than incomplete resection at an earlier age [7]. Several factors must be considered when determining the surgical indications, approaches, and extent of resection to minimize secondary complications when the inner ear structures are involved or the lesion is extensive [13].

Surgery is recommended when disease progression is aggressive and FD is accompanied by significant clinical symptoms, causing cosmetic head deformity, cranial nerve palsy, neurological symptoms, hearing loss, dysphagia, or breathing difficulty. The chosen operative procedure should focus on removal of diseased tissue, restoration of function, and prevention of complications [26].

Clear surgical indications are met in patients with progressive stenosis of the external auditory canal with accumulation of keratin and debris because this will inevitably lead to cholesteatoma formation and ear destruction, as in case 4. In such cases, most authors have recommended a primary canal wall down procedure because of the high chance of recurrence of the pathology 4 12. One study reported also successful trials of limited canalplasty 13. If middle ear involvement is present, tympanoplastic procedures should be attempted. However, as in two of our cases, although this seems to provide some hearing gain, the results are ultimately unsatisfactory; eventually, a hearing aid or bone-anchored system will probably be required in the majority of these cases.

Review of previously published studies demonstrated that 68% of children underwent surgical intervention, 21% were not treated surgically.

Two of our patients were qualified for surgical treatment. The treatment decision for the first patient was based on the presence of severe headaches unresponsive to medical therapy, cranial deformity, hearing loss, rapid disease progression, and the extent and location of the lesion. Although the cosmetic and anatomic results of surgery were satisfactory, hearing was unsatisfactory in spite of some functional improvement.

Before surgery in the most extensive case (case 2), we faced a management dilemma regarding whether to perform classic lateral petrosectomy with blind-sac closure of the external auditory canal or leave the canal open with the chance that hearing will improve. At the patient’s request, the auditory canal was left open after partial obliteration of the huge cavity with the temporalis muscle. After two reconstructive surgeries, the gain in hearing remained poor. In cases of canal occlusion, the best treatment choice is implantation of a bone-anchored hearing aid.

Bone-anchored systems are not precluded in patients with FD [6]. Failure of osseointegration is more likely to occur due to pathological bone transformation and osteointegration issues. Thus, the highest-quality bone should be checked for implantation. Transcutaneous bone-anchored system for hearing improvement has been used in one patient from the review and in the patient 4 from our series with good result. In this situation, leaving the canal open for implantation of a hearing aid is also not the worst option if use of a hearing aid will be finally chosen for treatment.

The second patient in the present study was qualified for surgery because of the presence of a complication of FD (mastoiditis and a cholesteatoma), and the surgery was a life-saving procedure. Canal wall down mastoidectomy was implemented with a good anatomic result and no disease recurrence.

Pardo-Maza et al. [11] described a case in which temporal bone FD developed 7 years after middle ear surgery involving cholesteatoma removal. This case emphasizes the importance of close follow-up after otologic surgery. Additionally, according to the literature, surgical treatment after adolescence results in better clinical outcomes and is associated with a lower intraoperative risk and more predictable clinical course of the disease. Independent from the time of surgical intervention, the possibility of disease recurrence should be kept in mind [7, 27]. The optimal treatment is total removal of the diseased tissue with simultaneous use of autogenic bone grafts for reconstruction if necessary. Some authors emphasize the importance of multidisciplinary team treatment [28].

During the last decade, medical treatment has played an increasingly important role in the management of FD in children. Several authors have reported their experience with two main nonsurgical treatments: bisphosphonates and monoclonal antibodies. However, the effectiveness and safety of these treatments in children remain controversial [29–32].

Bisphosphonates inhibit osteoclast activity with reported improvements in pain, bone turnover markers, lesion size (i.e., reduction or stabilization), and, in some studies, the radiographic appearance of FD lesions. Adverse effects include acute-phase reactions associated with the initial dose, which can manifest as fever, myalgia, or gastrointestinal upset [29]. Hypocalcemia caused by osteoclast inhibition is another possible adverse effect, as is a severe but very rare complication: osteonecrosis of the jaw. Furthermore, long-term suppression caused by bisphosphonates may result in serious consequences for the growing skeleton; this is an immensely important issue when treating children [29, 31, 32]. In 2004, Kos et al. [33] used pamidronate in six children with craniofacial FD with initially promising results as shown by pain relief and stabilization/reduction of the tumor size. The only reported adverse effect was fever. No long-term follow-up of this group regarding growth is yet available, however. In a 2014 review of bisphosphonates, Boyce et al. [29] also reported osteopetrosis and persistent bone remodeling defects associated with high doses and noted that the long-term effects of bisphosphonate treatment on skeletal healing have not been well studied in children.

Treatment with monoclonal antibodies includes mainly denosumab, which acts through inhibition of the receptor activator of nuclear kappa-B ligand. As reported by Eller-Vainicher et al. [30], treatment with denosumab resulted in rapid and complete disappearance of pain and bone turnover suppression. Denosumab reportedly has a better biochemical and clinical response than do bisphosphonates, which can be explained by the differences in their mechanisms of action. Thus, denosumab may represent a new treatment option; however, its long-term safety in children remains uncertain due to the lack of reports on the risk to linear growth and bone modeling and the few reports on fracture healing. Preclinical studies on animals have demonstrated an inhibitory effect on growth and the occurrence of osteopetrosis [30, 31].

In pediatric patients undergoing antiresorptive therapy, particular attention should be given to adverse effects and their impact on the child’s linear growth and development. In general, such medical therapy seems promising; however, the dosage, treatment period, and consequences of bone turnover suppression on the growing skeleton are not well defined. Because medical management can result in severe lifelong developmental consequences, treatment decisions should be made with consideration of the potential risks and benefits for each individual patient. We assume that antiresorptive treatment may be justified in altering the disease course in older children, in patients with severely disabling disease when no other treatment is effective, in patients with severe bone pain, and when surgical intervention is not possible. We have not met these criteria even in patients with progressive inner ear disease with symptoms of recurrent vertigo, moderate headache, and a high risk of unilateral hearing loss after an episode of temporary sudden deafness.

After consultations with the Department of Endocrinology of our institution, there was firm refusal to initiate medical treatment in the four herein-described children because of all of the abovementioned dangers.

## Conclusions

FD is a rare, benign disease in which symptoms are secondary to pressure and expansive growth of the lesion. The disease has various clinical courses that should be considered in the treatment decision-making process, and management decisions and treatment options should thus be proposed individually for each patient.

No therapeutic guidelines for FD are available. Generally, a “wait-and-scan” policy is an acceptable approach for most pediatric patients for whom functional and cosmetic problems are not prominent. Considering the tendency for FD to stabilize after adolescence, surgical treatment in children should be delayed until puberty.

In patients with external auditory canal stenosis, a canal wall down procedure should be performed earlier. When middle ear involvement is noted, a tympanoplastic procedure should be attempted. As illustrated in our cases, however, the results seem to be unsatisfactory due to further development of the pathology in the middle ear. In patients with cranial nerve deficits, severe headache, progressive hearing loss, or severe vertigo in which the involvement precludes the possibility of surgical treatment (e.g., the inner ear is affected), medical treatment can be implemented. However, the adverse effects of contemporary anti-FD drugs should be thoroughly discussed with the patients and their families. After reviewing the literature regarding temporal bone and general craniofacial FD, the general impression is that the differences in the clinical presentation of FD between adults and children are not substantial. Generally, surgery should be performed after puberty, and growth inhibition and adverse effects on growth in childhood should be considered when planning medical treatment.

## Abbreviations

CT: Computed tomography
EAC: External auditory canal
FD: Fibrous dysplasia
FN: Facial nerve
HL: Hearing loss
MRI: Magnetic resonance imaging
SNHL: Sensorineural hearing loss
